# Perspectives of Science in Iran

**Published:** 2010

**Authors:** Mohammad Abdollahi

It cannot be ignored that Iran had an impressive history in science over the very ancient years; through significant number of unique inventions, ideas, and discoveries brought by Iranian scientists such as Farabi, Toosi, Razes, Avicenna, Hesabi, and many others. During centuries due to political, cultural circumstances and war, Iran’s activities in all aspects of science and technology (S & T), as well as art have been changed. Expansion of internet and information technology in the last 50 years has eased estimation of impact of each scientist or institute or even countries in the world. When one examines scientometric databases such as Thompson Reuters (ISI). Scopus, or Google Scholar, the growth in Iranian S&T especially during the last 20 years is very evident. The S&T growth is seen in several fields such as chemistry, clinical medicine, pharmacology/toxicology/pharmaceutical sciences, physics, computer sciences, and engineering. The number of registered inventions and patents have also risen during the last 5 years. Regarding S&T, current data indicate that Iran is ranked after Turkey among its neighbors and the eighth in the Asia after Japan, China, India, Korea, Taiwan, Hong Kong, and Singapore. When one thinks about reasons of S&T growth in Iran, some major elements seem to be involved; for instance, attention of decision and policy makers to S&T in terms of research budget, training of experts, and updating instruments. Currently, Iranian government allocates around 0.5% of its GDP to S&T, which still ranks it behind industrialized countries which spend around 1.4% of their GDP on average. 

Despite such growth in S&T, brain drain has been increased in the last few years that need special attention. Regarding high potential of Iranian educated people, most of Iranians have got the best scientific jobs in the world especially in USA and other Western countries. Therefore, decision makers should be aware of establishing encouragement protocols to prevent brain drain. Some countries in the world, even the neighbor countries, have been lucky in returning their eminent scientists even those who had spent most of their lives abroad. The reality of the matter is that the brain drain is not an issue of only the developing countries. Lots of developed countries are dealing with a huge brain exodus especially to US, Canada, and Australia. Lessons should be learned from the countries that were having problems with brain drain and now not only they have overcome the issue, but also eventually have become “head hunters”. A good example is Canada! Dealing with up to 27% brain drain in 1920 and now absorbing scientists from every corner of the world. Technical Service Council was one of their strategies to deal with the problem.

Regarding brain drain in Iran, some solutions can be taken into account as follow: establishment of a national academy of genius scientists to create a cluster, establishment of knowledge business developing centers to hire scientists, emphasis on innovation and self-reliance, focusing in international collaboration and offering scholarships not only to eligible national graduate students but also to foreign young scientists and investing on them which will definitely contribute to knowledge transfer, considering changes in educational and examination systems and attempting to encourage innovation and problem solving skills in students, considering weekly/monthly scheduled meetings with students and getting updated in progress of their research, establishment of massive doctoral and postdoctoral training programs with cooperation of top universities of the world, revising eligibility 

criteria for appointments of faculty members and trying to hire tops of the world, closure of sub-standard institutions, encouragement of thinking scientifically among decision makers, working multiculturalism in scientific society, and finally upgrading the educational and research facilities to prevent time and budget waste. 

Ultimately, I would like to emphasize that one of Iran’s big potentials is its human resources, meaning intelligent young scientists. If good seeds are implanted today, then good fruits will be resulted in near future.

*Dr. Mohammad Abdollahi is currently working as a Professor of Toxicology and Pharmacology, Faculty of Pharmacy and Pharmaceutical Sciences Research Center, Tehran University of Medical Sciences, Tehran, Iran. He could be reached at the following e-mail address: mohammad@tums.ac.ir*

